# Soil Microbial Adaptation and Biogeochemical Feedback in Degraded Alpine Meadows of the Qinghai–Tibetan Plateau

**DOI:** 10.3390/microorganisms13051142

**Published:** 2025-05-16

**Authors:** Bingzhang Li, Quzhen Gesang, Yan Sun, Yuting Wang, Jibin Nan, Jun Xu

**Affiliations:** 1 Tibet Academy of Forest Trees, Lasa 851400, China; 2 School of Horticulture and Landscape, Yangzhou University, Yangzhou 225009, China

**Keywords:** alpine meadow degradation, soil physicochemical properties, metagenomic sequencing, microorganism community, biogeochemical cycling

## Abstract

Alpine meadows on the Qinghai–Tibetan Plateau are experiencing rapid degradation due to climate change and anthropogenic disturbances, leading to severe ecological consequences. In this study, we investigated the response of soil microbial communities and their metabolic functions across a degradation gradient using metagenomic sequencing and comprehensive soil physicochemical analysis in the city of Lhasa, China. Results showed that soil pH increased with degradation, while most nutrients, including different forms of nitrogen, phosphorus, and potassium, declined. pH, ammonium nitrogen, and organic matter were identified as key factors driving degradation dynamics. Microbial community composition shifted markedly, with distinct biomarker taxa emerging at different degradation levels. Network analysis revealed a progressive loss of microbial connectivity, with Actinobacteria dominance increasing in heavily degraded soils, while cross-phylum interactions weakened. Functional analysis of biogeochemical cycling genes showed that carbon, nitrogen, and phosphorus cycling were all disrupted by degradation, but each exhibited unique response patterns. These findings will extend our understanding of microbial-mediated soil processes under degradation and provide a scientific foundation for ecosystem management, conservation, and targeted restoration strategies in alpine meadows.

## 1. Introduction

The Qinghai–Tibetan Plateau is home to expansive alpine meadows that are critical for maintaining regional ecological balance, supporting biodiversity, and providing valuable ecosystem services [1]. However, these fragile ecosystems are experiencing rapid degradation due to a combination of climate change, overgrazing, and human activities [2]. Meadow degradation is a decline in vegetation cover, soil fertility, and overall ecosystem function. This process results in significant ecological and economic losses, including reduced carbon sequestration capacity, disrupted hydrological cycles, and diminished habitat quality for native species [3,4].

Soil is a cornerstone of alpine meadow ecosystems, serving as the foundation for plant growth and microbial activity, and a critical driver of ecosystem resilience [3]. Key soil parameters, such as pH, organic carbon content, and nutrient stoichiometry, are tightly linked to core ecosystem services, including primary productivity, carbon sequestration, and soil structural integrity [5,6]. In alpine meadows, where low temperatures constrain organic matter mineralization, even marginal losses of soil organic carbon can disproportionately impair water retention, microbial diversity, and plant community stability [7]. Chronic degradation disrupts critical symbioses (e.g., mycorrhizal networks), alters microbial community composition, and consequently impairs microbial functions such as nutrient cycling efficiency [8]. These changes lead to cascading effects, causing increased susceptibility to erosion, reduced forage quality, and diminished capacity to buffer climate extremes [9,10]. In addition, alpine soils under degradation often exhibit declining nitrogen and phosphorus pools due to erosion and disrupted plant–soil feedback, weakening their capacity to sustain vegetation cover and soil-binding root systems [11]. This bidirectional feedback loop, where soil degradation both drives and accelerates ecosystem decline, underscores soil’s dual role as a victim and catalyst of meadow collapse. Thus, quantifying soil physicochemical shifts is not merely diagnostic but predictive, offering insights into tipping points beyond which ecosystem recovery becomes implausible without intervention.

Soil microorganisms play critical roles in organic matter decomposition, nutrient mineralization, and biogeochemical cycling, serving as key drivers of ecological integrity and functioning of grassland ecosystems [12,13]. The degradation of meadow ecosystems introduces profound changes to microbial communities. Factors such as the loss of vegetation cover, reduced organic matter input, shifts in soil pH, and altered moisture regimes can dramatically influence microbial activity and community structure, ultimately intensifying soil degradation and accelerating ecosystem decline [14,15]. The relationship between soil degradation and microbial responses to it is complex and often contradictory in the literature. Some studies have reported sharp declines in microbial diversity and functionality, while others have observed shifts in dominant taxa or functional genes that suggest partial adaptation to degradation [1,8,16]. These discrepancies may arise from differences in environmental conditions, such as regional climate, soil type, and vegetation composition, as well as variations in the severity and duration of degradation. The disruption of microbial communities has far-reaching implications for grassland ecosystems. Beneficial microbes that promote plant growth and nutrient uptake may decline, reducing the efficiency of nutrient cycling and compromising plant health [17]. Conversely, the proliferation of opportunistic or pathogenic microbes can lead to plant stress, reduced biomass production, and increased vulnerability to disease [18]. These shifts in microbial dynamics often exacerbate soil nutrient loss, destabilize soil aggregates, and enhance erosion risks, further undermining the ecological balance of grasslands.

Understanding the interplay between microbial community profiles, soil physicochemical properties, and degradation severity is critical to unraveling the mechanisms driving alpine meadow collapse [1]. However, previous research has often examined these factors in isolation, while neglecting their intersectional dynamics [19,20]. Furthermore, key functional processes such as soil biogeochemical cycles driven by soil microorganisms, remain poorly studied due to previous technological limitations and era constraints. Metagenomic sequencing enables high-resolution profiling of both microbial taxonomy and functional gene content, providing critical insights into biogeochemical processes that cannot be fully captured by traditional amplicon-based methods. This study combines gradient-based field sampling across three levels of degradation (non-degraded, moderately degraded, and heavily degraded), detailed assessments of soil physicochemical properties, and high-resolution metagenomic sequencing to comprehensively investigate how soil microbial communities, their network structures, and key functional pathways related to biogeochemical cycling respond to alpine meadow degradation on the Qinghai–Tibetan Plateau. Specifically, we aim to (i) unravel the bidirectional feedback mechanisms linking soil physicochemical properties, degradation intensity, and microbial community structure; (ii) assess how microbial community composition and diversity shift across degradation gradients and evaluate their ecological roles in either buffering or intensifying soil degradation processes; (iii) elucidate microbial metabolic adaptations related to carbon, nitrogen, and phosphorus cycling across different degradation levels. The findings will offer a deeper mechanistic understanding of soil–microbe–plant feedback in degraded alpine meadows and provide valuable insight for developing targeted strategies to restore soil health and ecological function in degraded landscapes.

## 2. Materials and Methods

### 2.1. Sampling Information

The study was conducted in the city of Lhasa, China, located in the heart of the Qinghai–Tibet Plateau (29°38′–30°59′ N, 90°12′–91°45′ E). This region lies within the transitional zone between the temperate monsoon semi-humid climate and the subcold monsoon semi-arid climate of the plateau. The study area is situated at an altitude of approximately 4428 m above sea level, with an average annual temperature of 2.1 °C and an average annual precipitation of 617.5 mm. The dominant vegetation type in the region is alpine meadow. Sampling sites were selected based on field surveys that considered vegetation cover, aboveground biomass, and plant species composition. Sites were classified into non-degraded (ND), moderately degraded (MD), and heavily degraded (HD) meadows based on assessments of these three indicators [16]. Representative areas for each degradation level were chosen within the same general climatic context to minimize confounding variables. The non-degraded stage is characterized by abundant vegetation, high plant coverage (>85%), and substantial aboveground biomass, reflecting a healthy and minimally disturbed ecosystem. The moderately degraded stage is marked by a noticeable decline in vegetation density, with reduced plant coverage (40–60%) and biomass, suggesting moderate environmental degradation. The heavily degraded stage is defined by severely reduced vegetation cover (20–40%), low biomass, and sparse vegetation, indicating extensive degradation of the ecosystem [16].

Sampling was conducted as a one-time event in August 2024 to ensure consistency across sites and minimize seasonal variability. Seven samples were taken at each degradation level, resulting in a total of 21 samples. For each sampling site, geographic coordinates (longitude and latitude) were recorded. To evaluate vegetation characteristics, aboveground biomass (AGB), plant coverage, and plant diversity were assessed within four randomly placed 1 m × 1 m quadrats at each site. AGB was measured by harvesting all aboveground plant material within the quadrats, drying the samples at 80 °C for 48 h, and then weighing the dry biomass. Plant coverage was estimated visually as the percentage of ground area covered by live vegetation within the quadrats. Plant diversity was calculated using Simpson’s diversity index, which takes into account both species richness and the evenness of species distribution. Following the vegetation assessments, soil samples were collected from the topsoil layer (0–20 cm) at each site using a five-point composite sampling strategy. Specifically, at each 1 m × 1 m quadrat, soil cores were extracted from the four corners and the center using a sterile soil auger. The five cores were then thoroughly homogenized in the field to form a representative composite sample for that plot. The composite sample was then divided into two portions. One portion was air-dried, sieved through a 2 mm mesh, and stored for subsequent analysis of soil physicochemical properties. The second portion was immediately stored at −80 °C for later DNA extraction and microbial community analysis. Geographic information of the sampling locations was provided in Figure S1 and Table S1.

### 2.2. Soil Physicochemical Properties Analysis

Soil pH was measured in a 1:2.5 (*w*/*v*) soil-to-water suspension using a calibrated pH meter (PH700-BC, Apera, Shanghai, China). Organic matter (OM) and total nitrogen (TN) were quantified via an elemental analyzer (FlashSmart, Thermo Fisher, Waltham, MA, USA), following the standard procedures of drying and grinding the soil samples to a fine powder. Total phosphorus (TP) and total potassium (TK) were determined after acid digestion using ICP-MS (8900 Triple Quadrupole, Agilent, Santa Clara, CA, USA). Ammonium nitrogen (NH_4_-N) and nitrate nitrogen (NO_3_-N) were extracted with 2 M KCl, and their concentrations were measured colorimetrically, employing the indophenol blue method for NH_4_-N and the cadmium reduction method for NO_3_-N [21]. Available phosphorus (AP) was determined using the Olsen method, with extracts analyzed colorimetrically via the molybdenum blue method [22]. Available potassium (AK) was extracted using ammonium acetate and measured using flame photometry (FP910, PG Instruments, Leicestershire, UK) [23].

### 2.3. Soil DNA Extraction and Metagenome Sequencing

Soil DNA was extracted using the E.Z.N.A.^®^ Soil DNA Kit (Omega Bio-Tek, Norcross, GA, USA) following the manufacturer’s protocol. DNA purity and concentration were assessed using a spectrophotometer (NanoDrop, Thermo Fisher, Waltham, MA, USA) and agarose gel electrophoresis. High-quality DNA samples were used to construct metagenomic sequencing libraries. Libraries were prepared using a commercial library preparation kit (NEBNext^®^ Ultra™, New England, Ipswich, MA, USA) to ensure compatibility with Illumina sequencing platforms. The library preparation involved DNA fragmentation, end-repair, adapter ligation, and PCR amplification steps to create paired-end libraries with an average insert size of approximately 300 bp. Sequencing was performed on an Illumina platform with 150 bp paired-end reads, generating at least 20 Gb of data per sample. The raw reads obtained in this study were submitted to the National Center for Biotechnology Information (NCBI) under the accession number PRJNA1242428.

The raw metagenomic data were processed using EasyMetagenome, a pipeline established by Bai et al. [24], designed to streamline quality control, taxonomic profiling, assembly, and functional annotation. Briefly, raw sequencing data were processed using KneadData (v0.12.1) to remove low-quality reads and potential contaminants. Quality control included trimming and filtering with Trimmomatic, applying the following parameters: adapter removal using ILLUMINACLIP with seed mismatches = 2, palindrome clip threshold = 40, and simple clip threshold = 15; SLIDINGWINDOW trimming (4:20); and discarding reads shorter than 50 bp (MINLEN:50). After quality filtering, high-quality reads from all samples were pooled for de novo assembly using MEGAHIT (v1.2.9). Prodigal (2.6.3) was then used to identify open reading frames (ORFs) within the assembled contigs. CD-HIT (v4.8.1) was then applied to cluster highly similar sequences and produce a non-redundant gene set for subsequent analyses. Gene abundance was quantified using Salmon (v1.10.1), which aligns reads to the non-redundant gene catalog to estimate the relative abundance of each gene across all samples. Functional annotation focused on biogeochemical cycles was conducted by aligning the identified genes against curated reference databases. Genes involved in carbon, nitrogen, and phosphorus metabolism were specifically annotated using the CAZyDB [25], NCycDB [26], and PCycDB [27] databases, respectively. Taxonomic classification was performed using Kraken2 (v2.1.4), a k-mer-based tool, and results were refined using Bracken (v2.9) to generate more accurate abundance estimates of microbial taxa at various levels. To ensure fair comparisons of community diversity across samples, rarefaction was applied to the taxonomic abundance table using the vegan package in R prior to diversity and compositional analyses. Community similarity was assessed using principal coordinate analysis (PCoA) based on Bray–Curtis dissimilarity, which allowed the visualization of differences in microbial composition between samples. Shannon diversity and Chao1 richness indices were calculated using the vegan package in R. Differentially abundant taxa were identified using LEfSe (Linear Discriminant Analysis Effect Size), which provided insights into the most significantly enriched microbial taxa in each sample group [28].

### 2.4. Network Analysis

A co-occurrence network was constructed using the top 500 most abundant microbial species. Spearman’s correlation coefficients were calculated for all possible pairs of species. Correlations with a coefficient greater than 0.85 and a false discovery rate (FDR)-adjusted *p*-value of less than 0.05 were considered statistically significant and included in the network [29]. The resulting network was visualized in Gephi (v0.10) using the Fruchterman–Reingold algorithm, which optimizes the spatial arrangement of nodes to improve interpretability and highlight patterns of co-occurrence.

To further analyze the network structure, a Zi-Pi analysis was conducted using the igraph package in R (v4.4.1). In this framework, microbial taxa (nodes) are classified into four categories based on their within-module degree (Zi) and among-module connectivity (Pi). Specifically, network hubs serve as central and highly connected nodes, influencing the overall structure of the network. Module hubs are highly connected nodes within their specific modules but may not have extensive connections across the network. Connectors link different modules, facilitating inter-module communication. Peripherals are nodes with limited connections, mostly localized within their respective modules, and play relatively minor roles in the broader network. This classification provides a deeper understanding of microbial community organization, highlighting key taxa that shape co-occurrence patterns and potential ecological interactions [30].

### 2.5. Statistical Analysis

Statistical analyses were conducted using IBM SPSS Statistics (v26.00). Differences among groups were assessed using analysis of variance (ANOVA) with a significance threshold set at *p* < 0.05. Unless otherwise specified, data visualization was performed in R (v4.4.1). A random forest analysis was applied to rank the soil physicochemical factors influencing meadow degradation utilizing the rfPermute package (v2.5.4). Variable importance was determined based on the increase in mean squared error (IncMSE), and results were visualized to highlight the most influential factors. Partial dependence plots were then generated to illustrate how specific physicochemical factors influenced the degree of meadow degradation [31].

## 3. Results and Discussion

### 3.1. Changes in Vegetation and Soil Properties Across Meadow Degradation Levels

**Figure 1:**
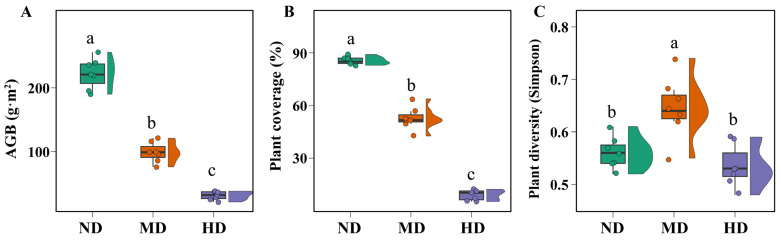
Vegetation characteristics of alpine meadows at different degradation levels. Different lowercase letters indicate significant differences at *p* < 0.05. Abbreviations: ND—non-degraded meadow; MD—moderately degraded meadow; HD—heavily degraded meadow. (**A**) Aboveground biomass; (**B**) Plant coverage; (**C**) Plant diversity (Simpson).

The vegetation exhibited distinct differences across the various levels of degradation in the alpine meadow (Figure 1). Consistent with expectations, both aboveground biomass (AGB) and plant coverage declined significantly as degradation intensified (Figure 1A,B). Specifically, AGB decreased by 55.39% and 86.04% in the moderately and heavily degraded meadows, respectively, while plant coverage declined by 38.06% and 76.96%. Notably, plant diversity followed a different trend. The Simpson diversity index was highest in the moderately degraded meadows, whereas the non-degraded and heavily degraded meadows showed no significant differences in diversity (Figure 1C). This pattern may be attributed to the intermediate disturbance hypothesis [32]: moderate degradation reduces the dominance of competitive species, thereby creating niches for subordinate species and increasing overall diversity. In contrast, severe degradation likely reduces species richness by impairing the environment’s capacity to support both dominant and subordinate species, while non-degraded meadows maintain stable but relatively uniform species compositions.

**Figure 2:**
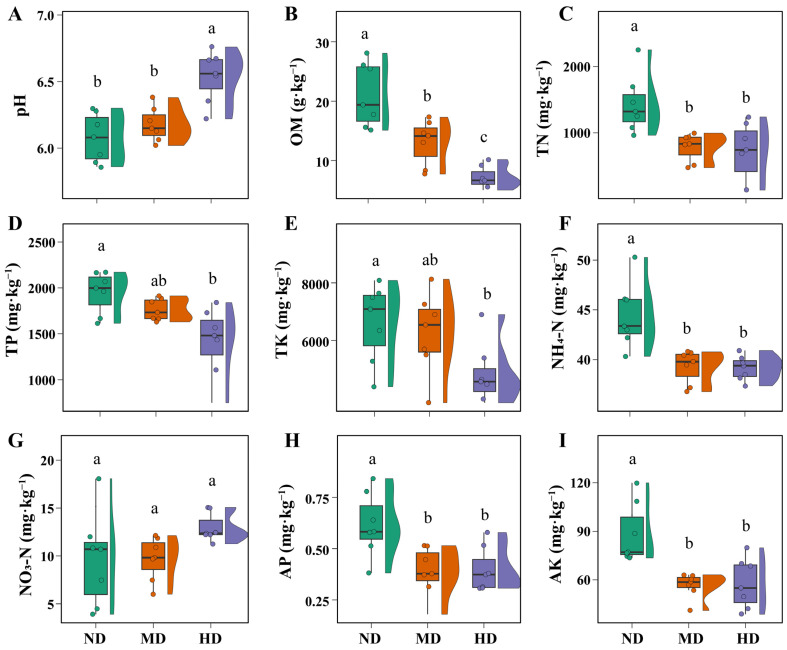
Soil physicochemical properties of alpine meadows at different degradation levels. Different lowercase letters indicate significant differences at *p* < 0.05. Abbreviations: ND—non-degraded meadow; MD—moderately degraded meadow; HD—heavily degraded meadow. (**A**) pH; (**B**) Organic matter (OM); (**C**) Total nitrogen (TN); (**D**) Total phosphorus (TP); (**E**) Total potassium (TK); (**F**) Ammonium nitrogen (NH_4_-N); (**G**) Nitrate nitrogen (NO_3_-N); (**H**) Available phosphorus (AP); (**I**) Available potassium (AK).

The primary soil physicochemical factors at each degradation level were measured to evaluate their role in the degradation process (Figure 2). The relationship between meadow degradation and soil physicochemical properties is not a simple one-way causal interaction; rather, the two mutually reinforce each other through a positive feedback loop, resulting in a downward spiral of ecosystem degradation. Among these factors, pH emerges as a particularly critical parameter that warrants closer attention. pH directly influences nutrient bioavailability, microbial community structure, and enzymatic activities, all of which are fundamental to soil health and ecosystem stability [33,34]. In moderately degraded meadows, a slight increase in pH was noted compared to non-degraded meadows, although this difference was not statistically significant. In heavily degraded meadows, however, pH exhibited a significant increase (Figure 2A). The result was consistent with the findings of previous study [1], which is likely driven by a combination of factors. The reduction in plant cover diminishes the release of acidic root exudates, which normally help maintain a lower pH in vegetated soils [35]. In addition, the decline of soil organic matter in degraded soil reduces the soil’s buffering capacity and limits its ability to maintain acidic conditions [36]. Such changes in pH have profound consequences for soil function, notably by altering nutrient bioavailability and disrupting microbial communities [33,34]. These disruptions may contribute to vegetation changes, including both reductions in plant cover and shifts toward species more tolerant of high-pH conditions, thereby exacerbating soil degradation. If left unchecked, these changes could intensify ecosystem instability and hinder natural recovery processes.

Soil nutrient levels exhibited a pronounced decline with increasing degradation. Compared to non-degraded meadows, organic matter content decreased by 37.85% in moderately degraded meadows and by 66.10% in heavily degraded meadows (Figure 2B). Similarly, total nitrogen levels dropped by 45.11% and 50.16% in these respective categories (Figure 2C). The observed decline in soil organic matter is likely attributed to multiple factors. Primarily, reduced vegetation cover limits the return of plant-derived organic residues, such as litter and root exudates, which are essential for maintaining nutrient pools [37]. Additionally, the degradation process may increase erosion and surface runoff, accelerating the physical loss of organic-rich topsoil [38]. Increases in soil pH can exacerbate these losses by disrupting microbial activity and enzymatic processes responsible for organic matter decomposition and nitrogen mineralization [39]. Furthermore, the decline of symbiotic plant species, particularly legumes associated with nitrogen-fixing bacteria, likely contributes to the reduction in biological nitrogen inputs [40]. The declines in total phosphorus (TP) and total potassium (TK) were relatively modest compared to carbon and nitrogen (Figure 2D,E). TP showed decreases of 9.60% and 27.40% in moderately and heavily degraded meadows, respectively, while TK declined by 5.30% and 29.97% in the same conditions. These slower rates of decrease may be attributed to the relatively stable nature of phosphorus and potassium in the soil matrix. Unlike organic matter and nitrogen, which are more directly linked to plant inputs and microbial activity, TP and TK are often bound to soil minerals and less prone to rapid losses [41,42]. As a result, their reduction occurs more gradually as degradation progresses. This trend aligns with previous studies, which have reported gradual reductions in TP and TK along degradation gradients [16], or relatively stable levels with slight declines depending on site-specific conditions [8].

In addition to total nutrient levels, soluble nutrients are equally important as they are directly available for uptake by plants and microorganisms. NH_4_-N, AP, and AK were significantly lower in moderately and heavily degraded meadows compared to non-degraded areas, but no significant differences were observed between the moderately and heavily degraded stages (Figure 2F,H,I). After moderate degradation, reduced vegetation resulted in decreased nutrient demand. Meanwhile, the soil microbial community exhibited a shift from K-strategists, which favor stable, resource-limited environments, to r-strategists, which rapidly exploit transient nutrient availability and thrive in disturbed conditions [43]. This transition may contribute to the slowing of nutrient consumption despite ongoing degradation. As nutrient demand from plants and microorganisms progressively declined, the activity of key soil enzymatic systems, such as alkaline phosphatase and urease, also decreased and eventually stabilized at a relatively low level. This stabilization suggests that under severe degradation, soil biochemical processes shift toward a functional baseline, maintaining only minimal nutrient cycling necessary for basic soil metabolic activity [44]. This likely stabilized nutrient mineralization rates at a lower threshold, resulting in a plateau in the decline of NH_4_-N, AP, and AK. This trend is consistent with previous findings [1,36], which also reported that soluble nutrient concentrations decline with increasing meadow degradation. Unlike other soluble nutrients, NO_3_-N content remained consistent across the three degradation levels (Figure 2G). The observed stabilization may be attributed to the effects of rising soil pH, which can stimulate the activity of ammonia-oxidizing archaea (AOA) and ammonia-oxidizing bacteria (AOB) [45,46]. Notably, AOA and AOB possess high substrate affinity and are capable of efficiently oxidizing low concentrations of ammonium [47,48]. This resilience of nitrifiers likely supports a basal level of nitrification activity even under nutrient-poor conditions. As a result, NO_3_^−^ concentrations remained relatively stable across the degradation gradient (Figure 2G), in contrast to the marked declines observed for other soluble nutrients.

**Figure 3:**
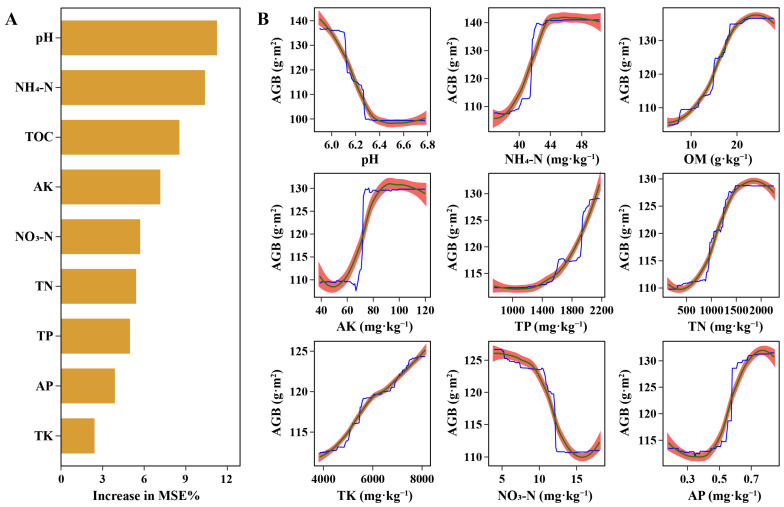
Influence of soil physicochemical properties on aboveground biomass. (**A**) Variable importance scores for soil physicochemical factors calculated by the random forest model; (**B**) partial dependence plots showing the relationships between individual soil physicochemical factors and AGB. In panel (**B**), the blue line represents the partial dependence estimates, the green line depicts a locally weighted polynomial regression trend, and the red shaded area indicates the 95% confidence interval. These partial dependence plots illustrate how changes in soil physicochemical properties affect the aboveground biomass.

The random forest model was then used to evaluate the relationship between soil physicochemical factors and meadow degradation levels. By using either AGB or plant coverage as a proxy for degradation, the results were similar (Figure 3 and Figure S2). Thus, we choose AGB as the representative metric of meadow degradation for this analysis. The model explained 77.41% of the variance in AGB, indicating a strong ability to capture the effects of soil properties on meadow degradation. Among the factors, pH, NH_4_-N, and organic matter content exerted the greatest influence on AGB, followed by TP, AK, and TN (Figure 3). All factors except pH demonstrated strong positive relationships with AGB, which highlighted the importance of nutrient availability in sustaining aboveground biomass. Interestingly, most nutrient factors exhibited S-shaped relationships with AGB. This suggests that while increases in these nutrients generally promote biomass production, the benefits diminish at higher concentrations, likely due to nutrient saturation effects or microbial competition [49]. This pattern underscores the complex dynamics of soil nutrient interactions and their nonlinear influence on plant productivity. In contrast, TP showed a distinct U-shaped relationship. As TP increased, AGB rose more rapidly, almost in a linear fashion. TP represents a larger, less immediately bioavailable nutrient pool that may gradually supply phosphorus over time. Therefore, the continuous availability of phosphorus from this pool supports a relatively steady increase in aboveground biomass without exhibiting a saturation plateau. These findings underline the need for balanced management of soil pH and nutrient supply. Efforts to limit excessive increases in pH, such as the judicious use of weakly acidifying or pH-stabilizing amendments, can help maintain favorable soil conditions for nutrient availability and microbial activity [50]. Enhancing organic matter level by incorporating organic amendments or crop residues can improve soil structure and fertility [51]. In addition, careful management of nitrogen inputs, such as maintaining adequate but not excessive NH4-N levels, is crucial to support plant growth while preserving beneficial plant–microbe symbioses. Supplementing soils with organic nitrogen sources, which release nitrogen gradually without causing large spikes in available inorganic nitrogen, may offer an ecologically sustainable strategy to enhance nitrogen supply. These strategies aim to rehabilitate severely degraded meadows and guide them toward improved ecological function and nutrient stability.

### 3.2. Soil Microbial Community Succession Across Meadow Degradation Levels

**Figure 4:**
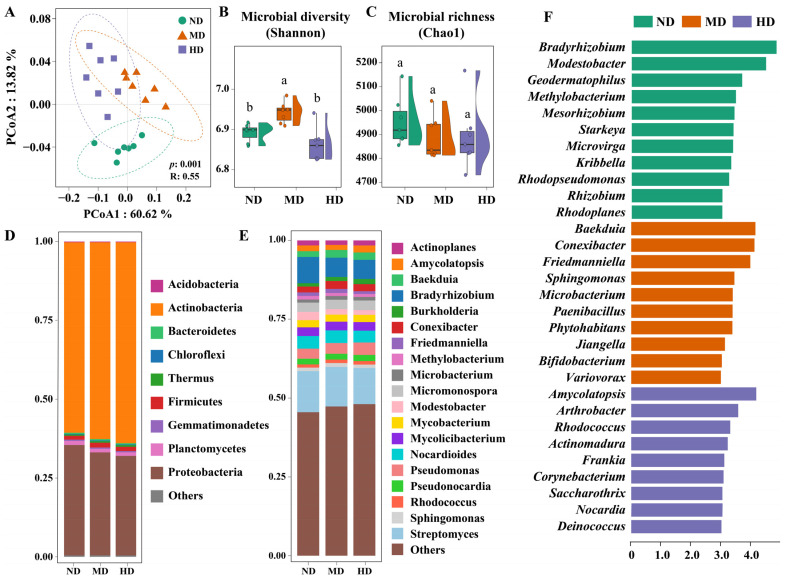
Microbial community profiles of alpine meadow soils at different degradation levels. (**A**) Principal coordinate analysis (PCoA) analysis based on Bray–Curtis distances, with ANOSIM results (R and *p* values) shown. (**B**,**C**) Microbial diversity indices including Shannon and Chao1, where different lowercase letters indicate significant differences at *p* < 0.05. (**D**,**E**) Taxonomic composition at the phylum and genus levels. (**F**) Key taxa enriched in different degradation levels as identified using LEfSe analysis.

The microbial community was significantly affected by alpine meadow degradation. PCoA analysis showed distinct microbial community composition among different degradation levels, a pattern confirmed by ANOSIM results (R = 0.55, *p* = 0.001) (Figure 4A). This variability likely arises because microbial communities are shaped by multiple, interrelated drivers. Three primary factors may be particularly important: (i) Plant community diversity and aboveground biomass will produce a variety of root exudates that interact with soil microorganisms, thus creating a dynamic and heterogeneous environment that shapes microbial composition [52,53]. (ii) Soil physicochemical properties such as nutrient levels and pH can significantly influence microbial populations [34,54]. These properties are also closely tied to the input and decomposition of plant litter, which supplies essential nutrients and alters soil chemistry over time. (iii) Microbial interactions themselves form another critical factor. As the meadow degradation progresses, changes in resource availability and environmental conditions lead to shifts in microbial populations, driving new ecological balances within the community [8]. These factors combine to create unique patterns of microbial community composition in alpine meadows. Specifically, the Shannon diversity index was higher in moderately degraded meadows, while non-degraded and heavily degraded meadows exhibited lower values without significant differences (Figure 4B). This may be explained by the partial loss of soil organic matter in moderately degraded meadows, combined with intermittent resource inputs (e.g., litter debris, root exudates) that create opportunities for niche differentiation [55]. In contrast, non-degraded and heavily degraded meadows experience a more homogenized ecological niche, leading to reduced species evenness. The Chao1 richness index, which measures species richness, did not show significant variation among the three groups (Figure 4C). This stability may be due to dormant opportunistic species becoming active during degradation, filling available niches and compensating for the loss of more sensitive species.

Actinobacteria and Proteobacteria dominated the soil microbial community, accounting for more than 94.3% of the total across all meadow degradation levels (Figure 4D). Other phyla such as Planctomycetes and Firmicutes only contributed approximately 5%. The microbial composition in these alpine meadow soils appeared relatively simple compared to more diverse soil environments, such as those found in temperate or tropical regions [56,57]. This limited diversity may be attributed to the harsh environmental condition characteristic of alpine meadows, including low temperatures, nutrient constraints, and challenging soil physicochemical properties, all of which can restrict the proliferation of a broader range of microbial taxa [58]. Notably, Actinobacteria displayed a pronounced increase in relative abundance as meadow degradation intensified. Prior studies have shown that Actinobacteria are particularly sensitive to soil pH, thriving in neutral to slightly alkaline conditions [16,59]. As degradation progresses, the associated gradual increase in soil pH likely provides a more favorable environment for Actinobacteria. Additionally, Actinobacteria’s ability to break down complex organic compounds and their adaptability to nutrient-poor environments likely contribute to their resilience and expansion under the altered resource availability and soil conditions accompanying alpine meadow degradation [60]. At the genus level, the dominant groups included *Bradyrhizobium*, *Burkholderia*, *Nocardioides*, *Mycobacterium*, *Streptomyces*, and *Pseudomonas* (Figure 4E). Among them, the K-strategists like *Bradyrhizobium* and *Streptomyces* exhibited a declining trend, while r-strategists such as *Pseudomonas* and *Burkholderia* increased. This shift likely reflects changing resource availability and environmental conditions associated with degradation. In these conditions, K-strategists, which thrive in stable environments with limited resources, are gradually outcompeted by r-strategists, which are better adapted to rapidly exploit transient nutrient inputs and flourish in more dynamic environments [43,61].

LEfSe was used to identify microbial biomarkers associated with each degradation level (Figure 4F). Non-degraded meadows are enriched with genera involved in nitrogen cycling and symbiotic plant interactions, with *Bradyrhizobium*, *Modestobacter*, and *Rhizobium* emerging as the distinctive taxa. The prevalence of nitrogen-fixing genera such as *Bradyrhizobium* and *Rhizobium* in non-degraded meadows aligns with their crucial role in maintaining soil fertility and plant resilience in healthy alpine ecosystems [62,63]. Their decline in degraded soils suggests disrupted nutrient cycling, potentially exacerbating plant loss and erosion. Moderately degraded meadows exhibited biomarkers linked to organic matter decomposition and degradation adaptation. *Baekduia* and *Conexibacte* dominated, alongside *Sphingomonas*, a genus known for metabolizing aromatic compounds [64]. Taxa like *Bifidobacterium* and *Variovorax* were also enriched. In moderate degradation, the enrichment of complex organic-degrading genera including *Sphingomonas* and *Conexibacter* may reflect increased recalcitrant carbon availability due to litter accumulation from declining vegetation [64]. Enrichment of *Bifidobacterium* and *Variovorax* could indicate shifts toward heterotrophic pathways in response to meadow degradation, though their roles in alpine systems warrant further study [65]. In heavily degraded soils, the enrichment of Actinobacteria such as *Amycolatopsis* reflects a pattern commonly observed in nutrient-poor and disturbed environments. These taxa frequently produce enzymes that mineralize soil organic matter, potentially accelerating carbon loss [66]. LEfSe analysis also identified pathogenic or opportunistic bacterial genera as significant biomarkers in these heavily degraded areas. For instance, *Corynebacterium* and *Nocardia* were significantly enriched, and both of them include species known to be pathogenic to plants and animals [67,68]. The co-occurrence of these pathogenic genera suggests a breakdown in beneficial plant–microbe interactions and an increase in soil toxicity, thereby establishing feedback loops that impair ecosystem recovery and contribute to the persistence of degraded conditions.

### 3.3. Microbial Interaction Patterns Across Meadow Degradation Levels

**Figure 5:**
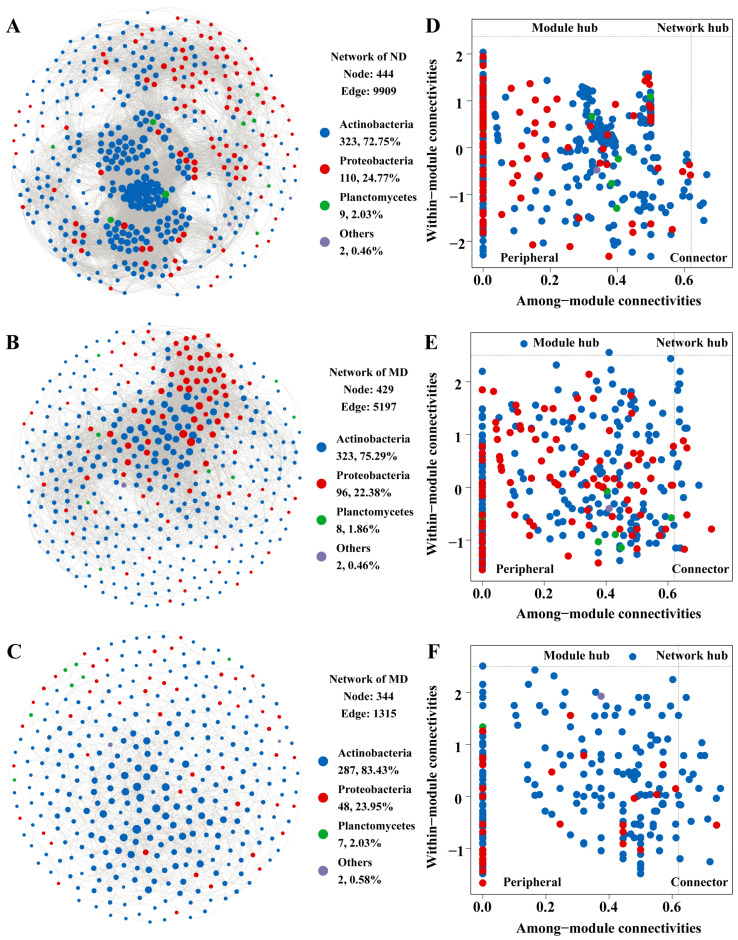
Network analysis of soil microorganisms across alpine meadows at different degradation levels. (**A**–**C**) Co-occurrence networks, with nodes representing microbial taxa and edges indicating strong and statistically significant interactions. Node colors correspond to taxonomic groups, and node size indicates the number of connections. (**D**–**F**) Zi-Pi plots showing different ecological roles of nodes. Microorganisms are categorized into four groups based on their within-module connectivity (Zi) and among-module connectivity (Pi).

To better understand how soil microbial communities respond to degradation, we analyzed co-occurrence network patterns across different meadow degradation levels (Figure 5A–C). Non-degraded meadows exhibited the highest complexity, with 444 nodes and 9909 edges. Moderately degraded meadows had 429 nodes and 5197 edges, while heavily degraded meadows were the most simplified, with only 344 nodes and 1315 edges. The average degree of the network also dropped steadily across the degradation gradient, from 44.64 in non-degraded to 24.23 in moderately degraded and 7.645 in heavily degraded meadows (Table S2). The marked decline in edge counts and average degree indicates a progressive loss of network connectivity. This fragmentation reduces the efficiency of microbial interactions, such as nutrient exchange and metabolic cooperation, which are crucial for maintaining soil health and ecosystem functions [69]. Graph density and clustering coefficients, which reflect network cohesion and local connectivity, also declined steadily as degradation progressed. The non-degraded area had the highest density (0.101) and clustering coefficient (0.57), followed by the moderately degraded area (density = 0.057, clustering coefficient = 0.52), and heavily degraded aera (density = 0.022, clustering coefficient = 0.42). In contrast, the network modularity, which reflects the degree of network compartmentalization, increased from 0.42 to 0.44 and 0.55 with meadow degradation, suggesting more distinct and isolated clusters at higher degradation levels. The dense and highly clustered network of non-degraded meadows reflects a well-connected microbial community that can sustain robust ecological processes. The decrease in clustering coefficient and graph density in moderately degraded meadows points to a network that, while still relatively intact, is beginning to lose coherence and efficiency [70]. By the time the system reaches heavily degraded meadows, the network structure becomes sparse and compartmentalized, as evidenced by the sharp reduction in edges and the rise in modularity. The progressive loss of microbial connectivity and rising network modularity under degradation may reflect a transition beyond the resilience threshold and lead to ecosystem destabilization as predicted by ecological network theory [71].

The analysis of edge composition highlighted key shifts in microbial relationships. Actinobacteria self-interactions accounted for 67.19% of all edges in non-degraded meadows. These interactions temporarily decreased in moderately degraded meadows (49.53%) before surging to 87.76% in heavily degraded meadows. Conversely, Actinobacteria–Proteobacteria interactions, which are critical for cross-phylum cooperation, peaked at 31.21% in moderately degraded meadows but dropped sharply to 8.97% in heavily degraded meadows. The extreme dominance of Actinobacteria self-interactions in heavily degraded meadows likely reflects their adaptation to nutrient-depleted environments. Actinobacteria are well suited to degrade recalcitrant organic matter, such as lignin and cellulose, which become relatively more dominant in the soil organic matter pool as plant biomass declines and easily decomposable substrates are depleted [51]. This adaptation enables them to form a self-reinforcing network that may exclude Proteobacteria through competitive interactions. The resulting disruption of cross-phylum synergies (e.g., carbon and nitrogen exchange) further weakens functional diversity. Similar patterns have been observed in desert soils, where stress-tolerant taxa form sparse, self-sustaining modules that dominate under extreme conditions [12]. Restoration efforts targeting moderately degraded soils could leverage the residual Actinobacteria–Proteobacteria synergy to rebuild cross-phylum functions. However, in heavily degraded soils, reintroducing Proteobacteria-linked taxa or organic amendments may disrupt Actinobacteria monopolies, reactivating labile carbon pathways.

The Zi-Pi analysis also revealed shifts in the structural roles of microbial taxa across the degradation gradient (Figure 5D–F). In the network of non-degraded soils, no module hubs or network hubs were present, and only four taxa were classified as connectors. The majority of taxa in this network were categorized as peripheral, indicating a decentralized network structure in non-degraded meadows, where most taxa play localized roles without a strong central influence [72]. In contrast, the network of moderately degraded soils showed emerging centralization, where two Actinobacteria taxa were identified as module hubs, signifying their increased structural importance within specific modules [29]. The number of connectors also increased significantly with contributions from both Actinobacteria and Proteobacteria. This shift reflects a trend toward a more centralized network structure, where certain taxa assume greater structural importance, likely due to their competitive advantage under moderate degradation [73]. The presence of connectors from multiple phyla suggests that cross-phyla interactions still occur, maintaining some degree of functional connectivity even under moderate degradation conditions. In the network of heavily degraded soils, although the total number of connector taxa remained comparable to that in moderately degraded meadows, their taxonomic composition changed markedly. Nearly all connectors in this stage belonged to the phylum Actinobacteria, with only one remaining taxon from Proteobacteria. This dominance suggests a decline in cross-phyla interactions, reflecting a contraction of the cooperative microbial network. Actinobacteria are known for their resilience under nutrient-poor and stressful conditions, often forming self-sufficient modules capable of decomposing recalcitrant organic matter [51]. However, the reliance on a narrow group of taxa reduces functional redundancy and limits the metabolic flexibility of the community. Furthermore, the compositional shift in connector taxa likely impairs microbial syntrophy or inter-species metabolic cooperation that facilitates complex nutrient transformations. In healthy soils, syntrophic interactions between Actinobacteria, Proteobacteria, and other phyla enable efficient cycling of carbon and nitrogen through complementary metabolic pathways. The loss of such cooperative links in heavily degraded soils may lead to inefficiencies in biogeochemical processes and a lower capacity to buffer environmental fluctuations. Combined with the continued absence of network hubs, which typically act as integrative nodes supporting system-wide connectivity, this pattern reflects a progressively fragmented and compartmentalized microbial community as degradation intensifies.

### 3.4. Soil Biogeochemical Cycles Affected by Degradation Levels

**Figure 6:**
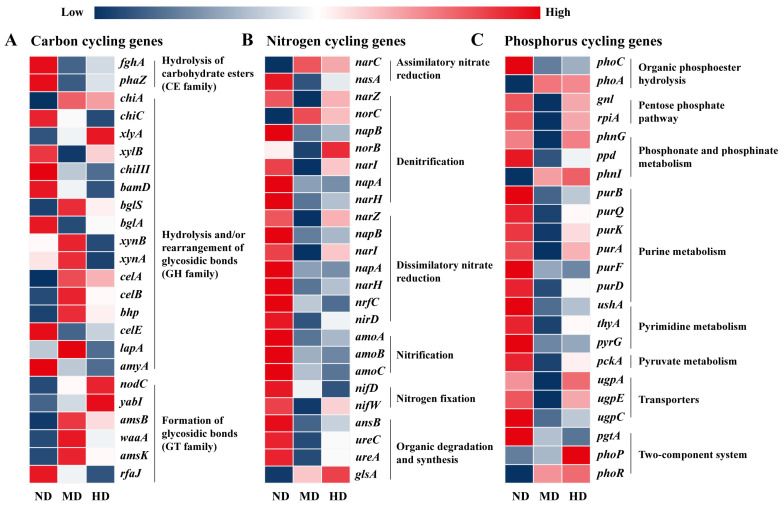
Heatmap depicting the relative distribution of functional genes involved in biogeochemical cycles that differ significantly across degradation levels. Panels (**A**–**C**) correspond to genes associated with carbon, nitrogen, and phosphorus cycling, respectively.

To elucidate how microbial functional adaptations influence soil health and ecosystem stability under environmental degradation, biogeochemical cycles including carbon, nitrogen, and phosphorus metabolism were further analyzed (Figure 6). In carbon cycling, cellulase-encoding genes such as *celA*, *celB*, and *bhp*, show an increase in moderately degraded soils, followed by a decline in heavily degraded soils. Similarly, the hemicellulase genes *xynA* and *xynB* exhibit a comparable pattern (Figure 6A). Moderate degradation would enhance litter input due to increased grass mortality, resulting in a transient influx of lignocellulose-rich substrates. This temporary lignocellulose subsidy likely drives the microbial community to upregulate cellulase and hemicellulase genes, enabling more efficient exploitation of this resource [74]. However, as degradation intensifies and transitions into heavily degradation, the loss of plant cover and further decline in litter production reduce the availability of cellulose and hemicellulose substrates. Residual carbon sources in heavily degraded soils may come primarily from microbial necromass or protected organic matter within soil aggregates, which may not require high levels of enzymatic activity. The chitin-degrading enzymes *chiA* and *chiC* exhibit divergent trends across the degradation gradient. As a multifunctional chitinase, *chiA* initially increases in moderately degraded soils. This rise is likely driven by fungal necromass resulting from disrupted plant–fungal symbioses, peaking as microbes exploit this transient resource. However, in heavily degraded soils, *chiA* declines but remains present at low levels, possibly targeting residual arthropod exoskeletons or microbial cell walls [75]. In contrast, *chiC*, a more specialized and less versatile chitinase, decreases steadily with increasing degradation. This decline is likely due to functional redundancy and energy trade-offs, as microbial communities in resource-limited environments favor high-efficiency enzymes like *chiA* [76]. These opposing trends highlight shifting carbon sources and the microbial community simplification that occurs with degradation, balancing the need to scavenge recalcitrant chitin against the metabolic costs of enzyme production.

Nitrogen cycling genes exhibit a distinct pattern from carbon-related genes in response to degradation (Figure 6B). Nearly all nitrogen cycling genes demonstrate a higher abundance in non-degraded meadows, with no consistent increasing or decreasing trends observed in moderately and heavily degraded soils. This variation could stem from the contrasting ecological and biogeochemical dynamics of nitrogen and carbon metabolism. In non-degraded meadows, the presence of robust plant cover and continuous organic matter inputs maintains a steady supply of nitrogen substrates, thereby supporting a range of nitrogen cycling processes such as nitrate reduction, nitrogen fixation, and nitrification. This stable environment fosters diverse microbial communities that contribute to a well-integrated nitrogen cycle [77]. However, as degradation progresses, this balance is disrupted. The loss of vegetation cover leads to reduced input of nitrogen-rich plant residues, while declining organic nitrogen in the soil limits the production of ammonium through mineralization. Nitrogen-fixing bacteria rely heavily on their interactions with living plants to carry out nitrogen metabolism effectively, such as root exudates and symbiosis [78]. The disruption of these plant–microbe relationships weakens nitrogen fixation pathways (e.g., *nifD*, *nifW*) and alters the composition of the microbial community. The overall effect is a decline in functional redundancy and a narrowing of nitrogen cycling pathways, resulting in a more fragmented nitrogen cycle in degraded soils. This pattern underscores the vulnerability of nitrogen cycling to ecosystem degradation. Restoring vegetation cover and organic matter inputs is therefore essential to reestablish a balanced nitrogen cycle. By fostering conditions that support nitrogen cycling microorganisms, conservation strategies can help stabilize soil fertility and promote long-term ecological resilience.

Phosphorus cycling genes also exhibit diverse patterns compared to nitrogen cycling genes in meadow degradation. While phosphorus-related genes are most abundant in non-degraded soils, they drop to their lowest levels in moderately degraded soils before showing some recovery in heavily degraded conditions (Figure 6C). This divergence arises from the distinct ecological roles and resource pools that nitrogen and phosphorus rely on. Nitrogen cycling heavily depends on plant–microbe interactions, including rhizosphere ammonium supply, which deteriorates irreversibly as vegetation and root systems decline. Phosphorus cycling, however, can leverage mineral-bound and recalcitrant organic phosphorus pools, allowing for a degree of functional recovery even under severe degradation. For instance, the *phoA* gene, which hydrolyzes phosphate from complex phosphoesters, exhibits increased abundance in heavily degraded meadows. Previous research has shown that *phoA* is strongly induced under phosphate-limiting conditions [79]. This pattern suggests microbial adaptation from relying on easily available inorganic phosphate to utilizing more recalcitrant organic phosphorus sources, such as phytate, phospholipids, and nucleic acids. Similarly, genes such as *phnI*, *phnG*, and *ppd*, which target phosphonates (C-P bond-containing compounds) [80], become more prevalent as degradation intensifies. The enrichment of these specialized phosphorus acquisition genes reflects a clear case of niche differentiation, where microbial taxa capable of utilizing rare or complex phosphorus sources are selectively favored under nutrient-stressed conditions. Moreover, the upregulation of the phoP/phoR two-component system, which governs the Pho regulon response under phosphorus starvation, highlights a coordinated community-level adaptation to long-term phosphorus scarcity [79]. These findings align with ecological theories suggesting that environmental stress selects for shifts in community functional traits, leading to greater functional specialization [81]. In this context, the increasing reliance on specialized phosphorus metabolism pathways indicates that microbial nutrient cycling becomes progressively constrained to a narrower range of strategies, which may reduce the flexibility and resilience of ecosystem processes under continued degradation.

## 4. Conclusions

This study highlights the impact of alpine meadow degradation on soil properties, microbial communities, and biogeochemical cycling. Degradation led to increased soil pH and nutrient depletion, with pH, NH_4_-N, and organic matter identified as key influencing factors. Microbial community composition shifted significantly, with a progressive loss of microbial connectivity as degradation intensified. Actinobacteria became increasingly dominant in heavily degraded soils, while cross-phylum interactions weakened. Functional analysis showed distinct responses among carbon, nitrogen, and phosphorus cycling genes, with nitrogen cycling being the most affected due to disrupted plant–microbe interactions. In contrast, microbial phosphorus acquisition partially recovered in heavily degraded soils through alternative pathways. These findings underscore the crucial role of microbial communities in ecosystem stability and nutrient cycling under degradation. Maintaining soil organic matter and nutrient availability while supporting microbial functional diversity may help mitigate degradation and enhance alpine meadow resilience. Future studies should explore long-term recovery dynamics and microbial-based conservation approaches.

## Data Availability

The original contributions presented in the study are included in the article, further inquiries can be directed to the corresponding author.

## Supplementary Materials

The following supporting information can be downloaded at: https://www.mdpi.com/article/10.3390/microorganisms13051142/s1, Figure S1: Sampling sites; Figure S2: Influence of soil physicochemical properties on plant coverage; Table S1: Geographic information of sampling site; Table S2: Topological indices of co-occurrence networks.
