# Supplementary material for: Soil Microbial Adaptation and Biogeochemical Feedback in Degraded Alpine Meadows of the Qinghai–Tibetan Plateau
Source: Microorganisms. 2025 May 16;13(5):1142. doi: 10.3390/microorganisms13051142 (PMC12114374; doi:10.3390/microorganisms13051142)
Supplement: Supplementary file 1 [file microorganisms-13-01142-s001.zip › microorganisms-3583857-supplementary.pdf]

Supplementary materials for

Soil microbial adaptation and biogeochemical feedbacks in degraded alpine meadows of the Qinghai-Tibetan Plateau

Bingzhang Li [a], Quzhen Gesang [a], Yan Sun [a], Yuting Wang [a], Jibin Nan [a], Jun Xu [a, b, *]

[a] Tibet Academy of Forest Trees, Lasa, Xizang 851400, China;

[b] School of Horticulture and Landscape, Yangzhou University, Yangzhou, Jiangsu 225009, China.

[*] Corresponding authors at: Tibet Academy of Forest Trees, Lasa, Xizang 851400, China; School of Horticulture and Landscape, Yangzhou University, Yangzhou, Jiangsu 225009, China.

E-mail address: 006963@yzu.edu.cn (J. Xu)

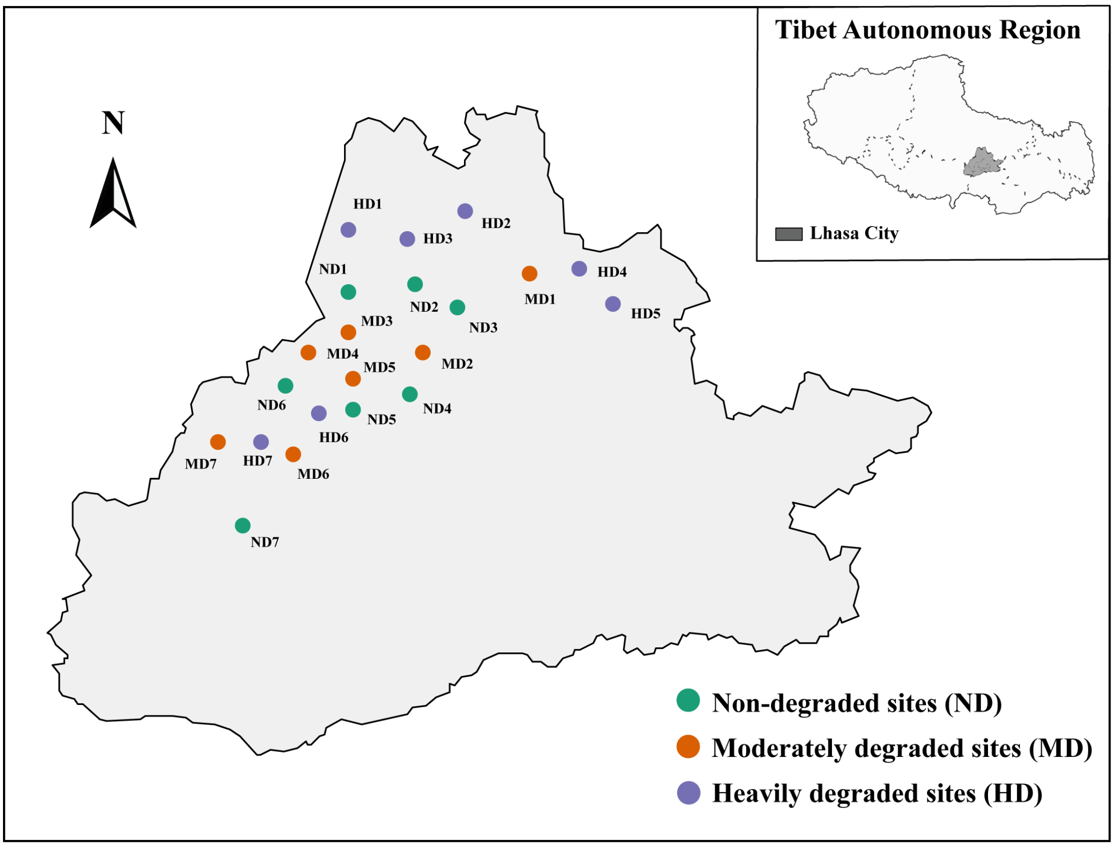

Figure S1 Sampling sites. A map showing the soil sampling sites of the alpine meadow across different degradation levels.

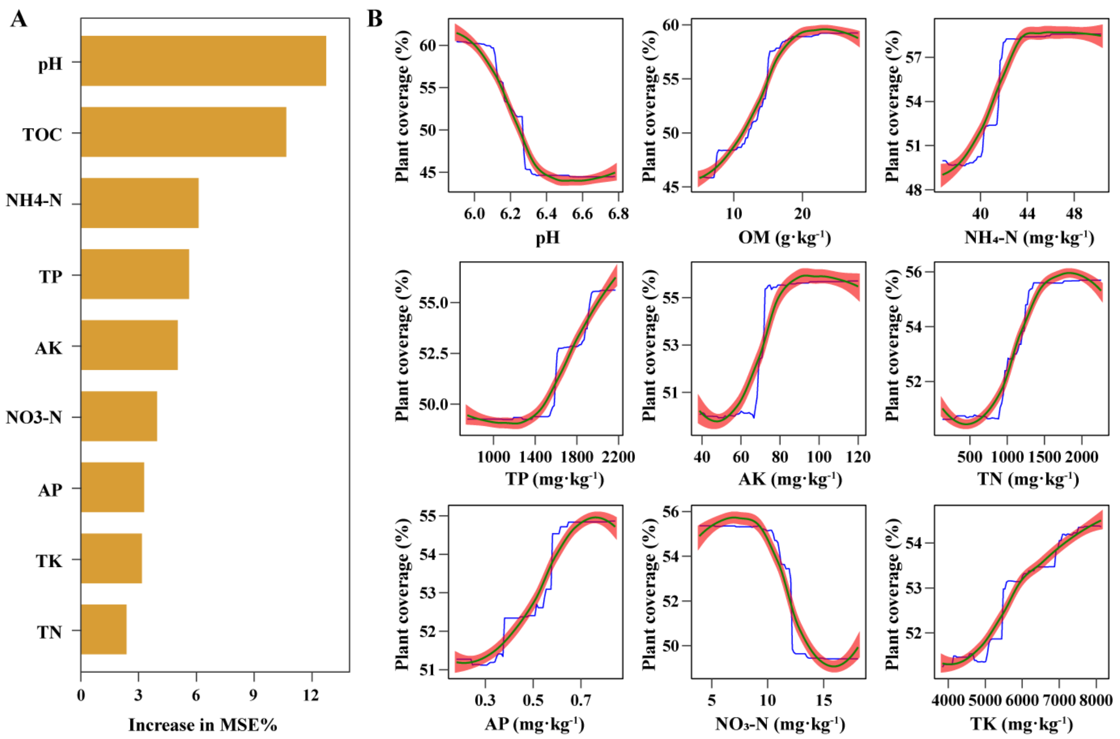

Figure S2. Influence of soil physicochemical properties on plant coverage. (A) Variable importance scores for soil physicochemical factors calculated by the random forest model; (B) Partial dependence plots showing the relationships between individual soil physicochemical factors and plant coverage. In panel B, the blue line represents the partial dependence estimates, the green line depicts a locally weighted polynomial regression trend, and the red shaded area indicates the 95% confidence interval. These partial dependence plots illustrate how changes in soil physicochemical properties affect the plant coverage.

Table S1 Geographic information of sampling site

		Longitude (E)	Latitude (N)
Non-degraded sites	ND1	90°42′	30°36′
	ND2	90°53′	30°36′
	ND3	91°1′	30°34′
	ND4	90°56′	30°15′
	ND5	90°46′	30°11′
	ND6	90°29′	30°16′
	ND7	90°24′	29°50′
Moderately degraded sites	MD1	91°18′	30°34′
	MD2	90°54′	30°24′
	MD3	90°42′	30°26′
	MD4	90°37′	30°22′
	MD5	90°44′	30°19′
	MD6	90°30′	30°4′
	MD7	90°16′	30°8′
Heavily degraded sites	HD1	90°44′	30°43′
	HD2	90°1′	30°47′
	HD3	90°52′	30°42′
	HD4	91°25′	30°37′
	HD5	91°34′	30°31′
	HD6	90°38′	30°10′
	HD7	90°23′	30°5′

Table S2 Topological indices of co-occurrence networks

	Parameters	ND	MD	HD
Node	Total	444	429	344
	Actinobacteria	72.75%	75.29%	83.43%
	Proteobacteria	24.77%	22.38%	13.95%
	Planctomycetes	2.03%	1.86%	2.03%
	Others	0.46%	0.46%	0.58%
Edge	Total	9909	5197	1315
	Actinobacteria - Actinobacteria	67.19%	49.53%	87.76%
	Actinobacteria - Proteobacteria	21.95%	31.21%	8.97%
	Actinobacteria - Planctomycetes	2.83%	1.04%	0.23%
	Proteobacteria - Proteobacteria	6.65%	17.05%	1.37%
	Proteobacteria - Planctomycetes	1.03%	0.33%	0.68%
	Planctomycetes - Planctomycetes	0.05%	0.06%	0.30%
	Others	0.30%	0.78%	0.69%
Network	Average degree	44.64	24.23	7.645
	Average path length	3.20	3.29	4.53
	Graph diameter	10	10	14
	Graph density	0.101	0.057	0.022
	Clustering coefficient	0.57	0.52	0.42
	Modularity	0.42	0.44	0.55
